# Albumin Paclitaxel Compared with 5-Penfluorouracil, Lobaplatin, and Albumin Paclitaxel Combined with 5-Penfluorouracil in the Treatment of Human Gastric Cancer Cell AGS Line Autophagy and Apoptosis

**DOI:** 10.1155/2022/6015877

**Published:** 2022-06-10

**Authors:** Xingzhen Cheng, Fang Yang, Yang Wang, Wei Nie, Adarsha Mahendra Upadhyay, Maolin Zhang, Qian Wang, Zhiqiang Yan

**Affiliations:** ^1^Department of Clinical Medicine, Guizhou Medical University, Guiyang 550004, Guizhou, China; ^2^Department of Medical Laboratory Science, Guizhou Medical University, Guiyang 550004, Guizhou, China; ^3^Department of Laboratory Medicine, Affiliated Hospital of Guizhou Medical University, Guiyang 550004, Guizhou, China; ^4^Department of Gastrointestinal Surgery, The Affiliated Hospital of Guizhou Medical University, Guiyang 550004, Guizhou, China

## Abstract

**Background:**

Gastric cancer is one of the most common malignant tumors in the world. Albumin paclitaxel (Nab-PTX) is a novel microtubule inhibitor with albumin as the carrier. Several clinical trials are underway in gastric cancer, but the autophagy mechanism of Nab-PTX on gastric cancer is still unclear. The autophagy and apoptosis effects of Nab-PTX compared with 5-pentafluorouracil (5-Fu) and lobaplatin (LBP) in gastric cancer are also unclear.

**Objective:**

This article will compare the effects of Nab-PTX, 5-Fu, LBP, and albumin paclitaxel + 5-pentafluorouracil (Nab-PTX + 5-Fu) on AGS cells from the perspective of autophagy and apoptosis, which is to provide new ideas and experimental evidence for gastric cancer.

**Method:**

(1) Experimental groups were control (Ctrl), Nab-PTX, 5-Fu, LBP, and Nab-PTX + 5-Fu. (2) CCK-8 assay was used to reflect cell viability and proliferation. (3) The flow cytometry was used to perform the 24-hour apoptosis and cell cycle of each group. (4) Western blot assay was used to investigate autophagy signal proteins LC3I/LC3II, LC3II/LC3I, SQSTM1/p62, Beclin-1, Atg12, Atg5, p-mULK1, p-AMPK, p-mTOR, and apoptosis signal proteins Bax and Bcl-2.

**Results:**

Nab-PTX, 5-Fu, LBP, and Nab-PTX + 5-Fu inhibited AGS cells' proliferation and arrested the cell cycle. At the same time, each group increased the apoptosis of AGS cells to various degrees (Nab-PTX + 5-Fu > Nab-PTX > 5-Fu > LBP, respectively). The experimental results showed that Nab-PTX and Nab-PTX + 5-Fu promoted autophagy and apoptosis of AGS cells. The comparison of Nab-PTX, 5-Fu, and LBP between groups revealed that 5-Fu inhibited autophagy and the expression of apoptosis protein Bax. In LBP, abnormal activation of autophagy downstream, blocking of autophagy flow, abnormal increase of ATG12, and increased expression of apoptosis protein Bax occurred. Further study found that the autophagy upstream mechanism is different.

**Conclusion:**

Nab-PTX, 5-Fu, LBP, and Nab-PTX + 5-Fu can inhibit cell proliferation, promote cell apoptosis, and induce the difference in autophagy expression. The autophagy difference of this antitumor drug may be related to its inducing apoptosis. Meanwhile, Nab-PTX has a better antitumor effect than 5-Fu and LBP in gastric cancer, and the combination of Nab-PTX + 5-Fu has more antitumor advantages.

## 1. Introduction

According to the Global Cancer Statistics in 2018, gastric cancer has become the fifth most frequently diagnosed cancer in the world (with more than 1,000,000 new cases) and the third leading cause of cancer death worldwide (with an estimated death toll of 783,000) [1]. The burden of gastric cancer is particularly severe in China, where the incidence of gastric cancer accounts for 42.6% of the global incidence and 45% of all gastric cancer-related deaths [2]. Although most early gastric cancer can be cured by endoscopic or surgical resection [3], the prognosis of advanced gastric cancer is still poor, and chemotherapy plays a critical role in advanced gastric cancer. Oral fluorouracil plus oxaliplatin is used as the first-line chemotherapy for unresectable advanced gastric cancer in Asia [4]. However, the resistance to classical chemotherapy drugs has become an inevitable problem [5]. Drug resistance has become a significant obstacle to the effective treatment of advanced gastric cancer [6], so it is urgent to develop new treatment ideas and directions for gastric cancer.

At present, among the new chemotherapy drugs in clinical trials for gastric cancer, albumin paclitaxel (Nab-PTX) and lobaplatin (LBP) have broad application prospects. Nab-PTX is a new water-soluble microtubule inhibitor derived from paclitaxel (PTX). Compared with PTX, Nab-PTX does not require special solvents and preuse of steroids to treat tumors [7]. Therefore, Nab-PTX has the potential advantages of fewer side effects and avoiding immunosuppression. At present, a large number of clinical trials of albumin paclitaxel in cancer treatment have shown that albumin paclitaxel has sound antitumor effects [7–10]. The drug resistance of oxaliplatin and cisplatin in cancer treatment cannot be ignored. The LBP, as a third-generation platinum alternative, is characterized by good water solubility, high antitumor activity, and a broad anticancer spectrum. However, it is only approved for the treatment of metastatic breast cancer, chronic myeloid leukemia, and small cell lung cancer in China at present [11, 12]. The current clinical studies on LBP in gastrointestinal malignancies show that lobaplatin is safe and effective [13, 14].

Autophagy aims to repurpose basic structures such as amino acids to generate new cellular components to suit its metabolic needs by degrading cytoplasmic contents, abnormal protein polymers, and excess or damaged organelles. According to the different binding modes of autophagy and lysosome, autophagy can be divided into macroautophagy, microautophagy, and chaperone-mediated autophagy [15]. The autophagy involved in this article is mainly macroautophagy (autophagy for short). At present, there is no conclusion about the relationship between autophagy and tumor and antitumor drugs. Studies have shown that autophagy is closely related to *Helicobacter pylori* infection, the occurrence and development of gastric cancer, chemotherapy and drug resistance of gastric cancer, and prognosis [16]. Autophagy is activated under stress conditions, such as cellular nutrient deprivation, hypoxia, oxidative stress, and infection. At this time, autophagy acts as a survival mechanism for cells. However, excessive autophagy activation can lead to cellular programmed sexual death [17]. Autophagy can modulate the tumor microenvironment by mediating secretion, degradation, and cell signaling modification. The tumor microenvironment refers to the occurrence, growth, and metastasis of tumors closely related to the internal and external environment of tumor cells. Compared with normal tissues, it is characterized by low oxygen levels, high lactate levels, extracellular acidosis, and reduced nutrients. In addition to the tumor microenvironment related to autophagy regulation, antiangiogenic and immune-targeted therapies also seem to have the potential to improve tumor therapeutic efficacy by altering the tumor microenvironment [18]. However, the autophagy mechanism of Nab-PTX in the treatment of gastric cancer is still unclear. Therefore, this study was conducted to investigate whether Nab-PTX induced apoptosis of human gastric cancer cells (AGS) through the autophagy signaling pathway and compared the differences in autophagy expression and apoptosis of gastric cancer cells induced by Nab-PTX, 5-FU, LBP, and Nab-PTX + 5-Fu.

## 2. Materials and Methods

### 2.1. Main Materials and Instruments

The RMPI 1640, PBS buffer, common pancreatin (containing EDTA), pancreatin without EDTA, and fetal bovine serum were purchased from Biological Industries (BI), and Pierce RIPA buffer was purchased from Thermo. The reagents required by RIPA Lysis buffer were purchased from Shanghai Biyuntian Biotechnology Co., Ltd., and the CCK-8 kit was purchased from Dojindo Laboratories. The Annexin V-FITC/PI, an apoptosis detection kit, was purchased from Becton, Dickinson, and Company, and the cycling kit was also purchased from Becton, Dickinson, and Company. The required antibody and article number are as follows: GAPDH (GeneTex GTX100118), SQSTM1/p62 （Abcam ab109012 ）, ATG12 (Abcam ab109491), Beclin 1 (Abcam ab207612), p-AMPK (Abcam ab133448), Bax (Abcam ab182733), LC3I/II (CST 12741), Atg5 (CST 12994), p-ULK1 (CST 5869), p-mTOR (CST 5536), and Bcl-2 (CST 4223), the secondary antibodies purchased from Pumei Biotechnology Co., Ltd., Western blot gel kits purchased from Shanghai Biyuntian Biotechnology Co., Ltd., ECL luminescence Nab-PTX was purchased from Jiangsu Hengrui Pharmaceutical Co., Ltd., 5-Fu was purchased from Tianjin Jinyao Pharmaceutical Co., Ltd., and LBP was purchased from Hainan Chang 'an International Pharmaceutical Co., Ltd. The flow cytometer model BD Canto II plus (Becton, Dickinson and Company) and the microplate reader model Thermo Scientific Varioskan Lux (Thermo Fisher Scientific) were also purchased. The electrophoresis instrument model DYY-6C(Beijing Liuyi Biotechnology Co., Ltd.). The ECL chemiluminescence and gel imager model GeneGnome specialized chemiluminescence imaging system were also purchased.

### 2.2. Cell Culture

The human gastric cancer cells' AGS lines were obtained from the Chinese Academy of Sciences Cell Bank. AGS cells were cultured in a complete medium of RPMI-1640 supplemented with 10% fetal bovine serum in humid air containing 5% CO_2_ and in a constant temperature incubator at 37°C. The cells could be subcultured until 80% growth was achieved.

### 2.3. Cell Proliferation Assay (CCK-8 Assay)

A cell proliferation experiment was carried out using a CCK-8 assay. AGS cells were plated in a 96-well plate at a density of 1 × 10^^4^ cells/well and were cultured in RPMI 1640 containing 10% FBS. When the cells reach 80%, remove the original culture medium, wash with PBS, and add Nab-PTX (concentration gradient: 0.1 *μ*mol/l, 0.2 *μ*mol/l, 0.4 *μ*mol/l); 5-Fu (concentration gradient: 1 *μ*mol/l, 2 *μ*mol/l, 4 *μ*mol/l); LBP (concentration gradient: 5 *μ*mol/l, 10 *μ*mol/l, 20 *μ*mol/l); Nab-PTX + 5-Fu (concentration gradient: 0.1 *μ*mol/l + 1 *μ*mol/l, 0.2 *μ*mol/l + 2 *μ*mol/l, 0.4 *μ*mol/l + 4 *μ*mol/l); after being cultured for 24 hours, 48 hours, and 72 hours, respectively, 10 ul of CCK-8 solution was added to each well. After incubation at 37°C for 1 hour, measure the absorbance at the wavelength of 450 nm with a microplate reader.

### 2.4. Flow Cytometry Analysis Assay

First, 2 × 10^5^ AGS cells in the logarithmic phase were inoculated in a 6-well plate, and when they grew to 80%, they were treated with Nab-PTX (0.2 *μ*mol/l); 5-Fu (2 *μ*mol/l); LBP (10 *μ*mol/l). The cells were cultured with Nab-PTX + 5-Fu (0.2 *μ*mol/l + 2 *μ*mol/l) for 24 hours, washed with precooled PBS, digested with 0.25% trypsin without EDTA, and collected after centrifugation. The apoptosis detection: 100 *μ*l of binding buffer was added to the collected cells. The cells were incubated with 5 *μ*l of Annexin V-FITC and 5 *μ*L of propidium iodide at the darkroom temperature for 15 min; then, 400 *μ*l of binding buffer was added and then detected by flow cytometry. The cell cycle detection process was as follows: collecting fine cells, adding precooled 70% ethanol for fixation, centrifuging and removing the supernatant, washing cells with precooled PBS, adding 500 *μ*l staining buffer, 25 *μ*l PI, and 10 *μ*l RNA enzyme, taking a water bath at 37°C away from light for 30 min, performing cycle detection, and evaluating the percentage of cell arrest at each cell cycle stage.

### 2.5. Protein Extraction and Western Blot Assay

First, 2 × 10^5^ AGS cells in the logarithmic phase were inoculated into 6-well plates and treated with Nab-PTX (0.2 *μ*mol/l) when they grew to 80%. The, 5-Fu (2 *μ*mol/l), LBP (10 *μ*mol/l), and Nab-PTX + 5-Fu (0.2 *μ*mol/l + 2 *μ*mol/l) were cultured for 24 h, the culture medium was removed, and the cells were washed by precooling with PBS. The cells were lysed with precooling lysis buffer containing 1% protease inhibitor (Thermo), and proteins were collected and denatured. The accumulated proteins were separated by electrophoresis to separate protein molecules of different sizes on the gel and then transferred to the PVDF membrane. Subsequently, a blocking solution was added for sealing. The membrane was incubated with a designated primary antibody. Then, a secondary antibody was added to check the expression of the target protein. Results exposure was performed by ECL chemiluminescence and gel imager. At the same time, the ImageJ software was used for gray value analysis. The above experimental workflow is as follows:
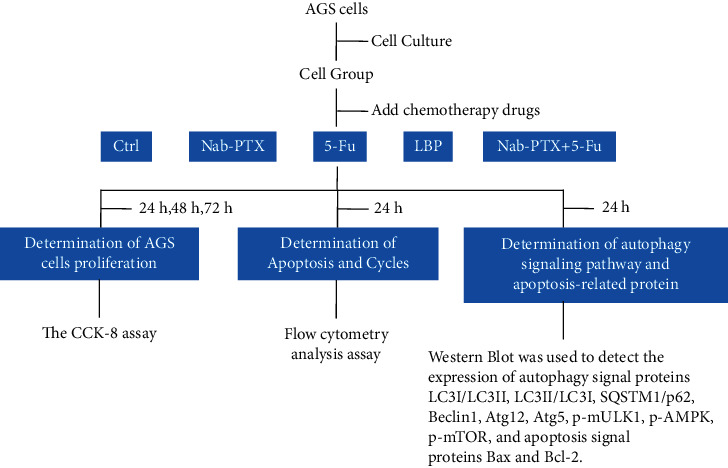


### 2.6. Statistical Analysis

SPSS25.0 statistical software was used for analysis. Mean ± standard deviation (*X* ± *S*) was used to represent all data, including at least three independent experiments. One-way ANOVA was used for multigroup comparison, and a *t*-test was used for pair comparison. *P* < 0.05 was considered statistically significant, *P* < 0.01 was considered statistically significant, and *P* < 0.001 was considered statistically significant.

## 3. Results

### 3.1. Nab-PTX, 5-Fu, LBP, and Nab-PTX + 5-Fu Can Inhibit the Proliferation of AGS Cells

**Figure 1:**
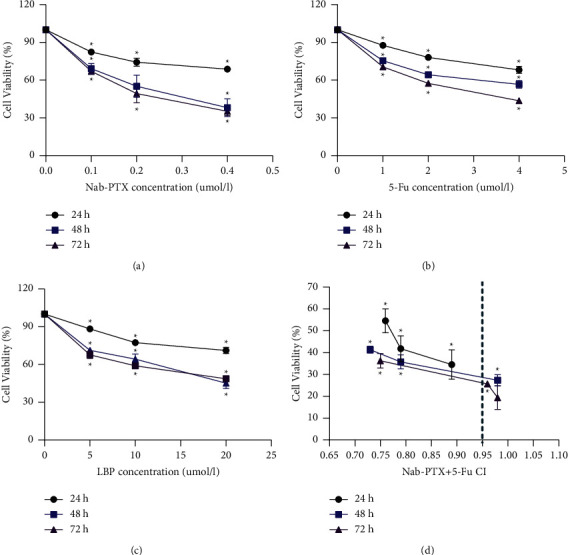
CCK8 assay to detect the cell inhibition rate of each group and each time period. (a) Nab-PTX (concentration gradient: 0.1 *μ*mol/l, 0.2 *μ*mol/l, and 0.4 *μ*mol/l) for 24, 48, and 72 h; (b) 5 -Fu (concentration gradient: 1 *μ*mol/l, 2 *μ*mol/l, and 4 *μ*mol/l) for 24, 48, and 72 h; (c) LBP (concentration gradient: 5 *μ*mol/l, 10 *μ*mol/l, and 20 *μ*mol/l) for 24, 48, and 72 h; (d) Nab-PTX + 5-Fu (concentration gradient: 0.1 *μ*mol/l + 1 *μ*mol/l, 0.2 *μ*mol/l + 2 *μ*mol/l, and 0.4 *μ*mol/l + 4 *μ*mol/l) for 24, 48, and 72 h . ^*∗*^*P* < 0.05 indicates statistical significance. Data are shown as mean ± SD of 3 wells, and it represents three independent experiments.

To determine the influence of each group on AGS cell proliferation, we performed CCK-8 cell proliferation detection and analysis. The results showed that compared with the control group (Ctrl), Nab-PTX, 5-Fu, LBP, and Nab-PTX + 5-Fu could effectively inhibit the growth of AGS cells. IC30 values of Nab-PTX at 24 h, 48 h, and 72 h are 0.34 *μ*mol/l, 0.095 *μ*mol/l, and 0.081 *μ*mol/l, respectively, as shown in Figure 1(a). For the control test, the IC30 values of single drug 5-Fu and LBP at 24 h, 48 h, and 72 h were 5-Fu, 3.5 *μ*mol/l, 1.5 *μ*mol/l, and 1.0 *μ*mol/l, respectively, and LBP was 19 *μ*mol/l, 5.9 *μ*mol/l, and 4.2 *μ*mol/l, as shown in Figure 1. We can find that the survival rate of each group decreases with the increase of action time, which is time-dependent.

### 3.2. The Apoptosis Rate of AGS Cells Treated with Nab-PTX + 5-Fu Was Higher Than That of Nab-PTX, 5-Fu, and LBP

**Figure 2:**
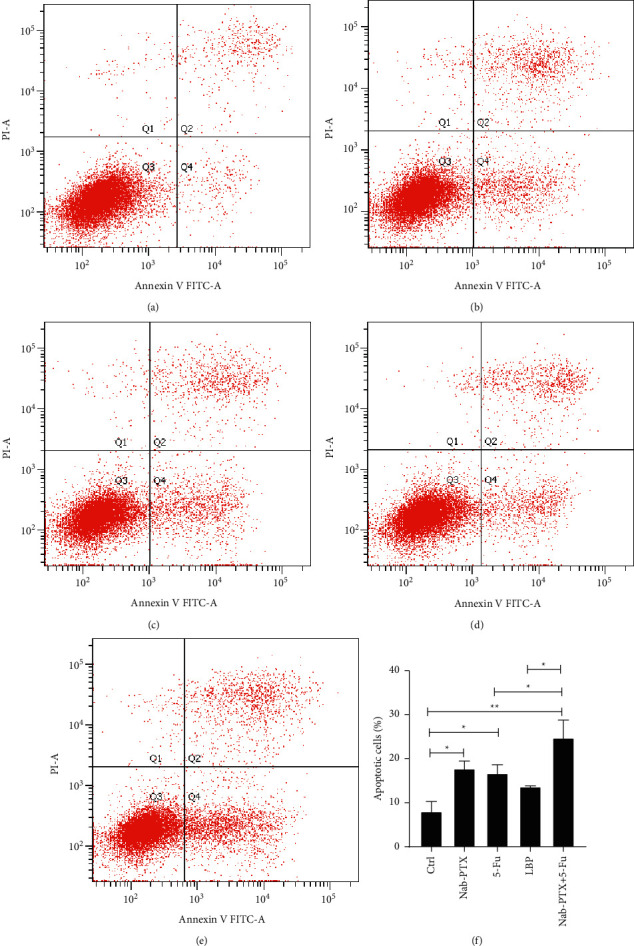
Apoptosis in each group of AGS cells. (a) The 24 h apoptosis map of control; (b) the 24 h apoptosis map of Nab-PTX(0.2 *μ*mol/l); (c) the 24 h apoptosis map of 5-F(2 *μ*mol/l); (d) the LBP(10umol/l) 24 h apoptosis map; (e) the 24 h apoptosis map of Nab-PTX + 5-Fu (0.2 *μ*mol/l + 2 *μ*mol/l); (f) apoptotic cells (%) in different groups, analyzed by Annexin V-FITC/PI and using flow cytometry, *Q*1 = mechanical damage, *Q*2 = late apoptotic cells, *Q*3 = live cells, *Q*4 = early apoptotic cells. All the above experiments were repeated at least three times independently. ^*∗*^*P* < 0.05, ^*∗∗*^*P* < 0.01. Nab-PTX, 5-Fu, LBP, and Nab-PTX + 5-Fu can block the cell cycle at different stages.

To compare the difference in apoptosis among Nab-PTX, 5-Fu, LBP, and Nab-PTX + 5-Fu, we performed Annexin V-FITC/PI flow cytometry, and the result showed that the apoptosis rate was 8.00 ± 2.50 in Ctrl (Figure 2(a)); 17.73 1.98 in Nab-PTX (Figure 2(b)); 16.67 ± 2.22 in 5-5-Fu (Figure 2(c)); 13.67 ± 0.40 in LBP (Figure 2(d)); 24.73 ± 4.31 in Nab-PTX + 5-fu (Figure 2(e)). Compared with the control group, the apoptosis rate of gastric cancer cells induced by each group was as follows: Nab-PTX + 5-Fu > Nab-PTX > 5-Fu > LBP.

**Figure 3:**
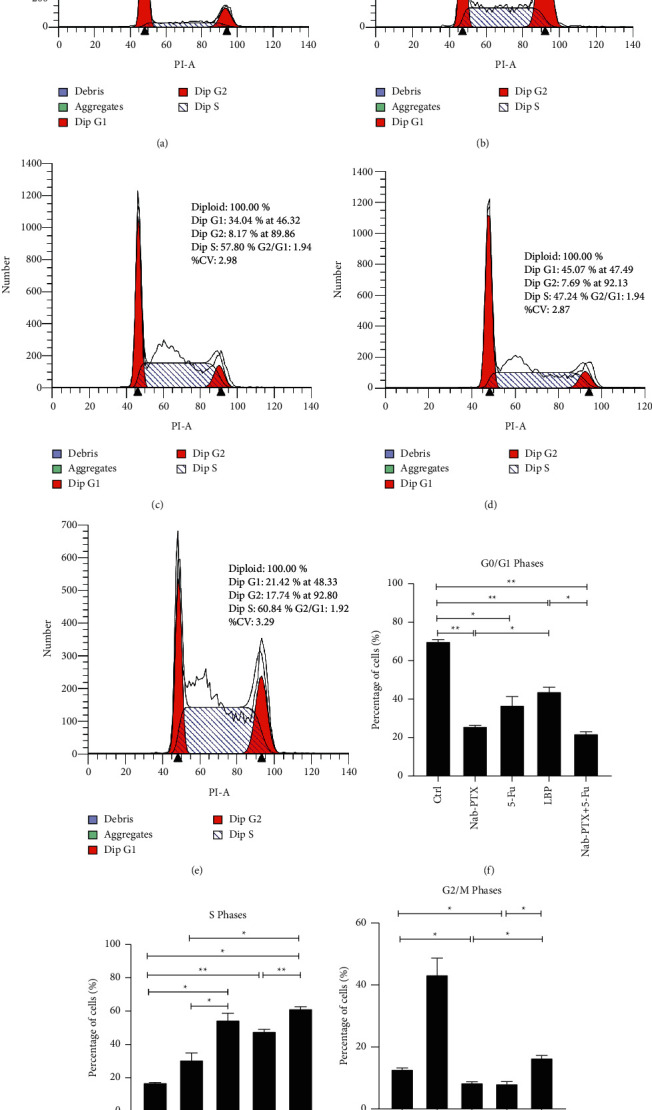
Cycle arrest in each group of AGS cells. (a) The 24 h cycle diagram of the control group; (b) the 24 h cycle diagram of Nab-PTX (0.2 *μ*mol/l); (c) the 24 h cycle diagram of 5-Fu (2 *μ*mol/l); (d) the 24 h cycle diagram of LBP (10 *μ*mol/l); (e) the 24 h cycle diagram of Nab- T + 5-Fu (0.2 *μ*mol/l + 2 *μ*mol/l); (f) Percentage of cells(%) in G0/G1 phases; (g) Percentage of cells(%) in S phases; (h) Percentage of cells(%) in G2/M phases, and the cell cycle was detected by flow cytometry. All the above experiments were repeated at least three times independently. ^*∗*^*P* < 0.05, ^*∗∗*^*P* < 0.01.

The flow cytometry cycle results were analyzed to study the potential mechanism of Nab-PTX inhibiting the growth of gastric cancer cells: the control group was mainly in G1/G0 phase (Figure 3(a)), the Nab-PTX group could arrest the AGS cell cycle in the G2/M phase (Figure 3(b)), and both the 5-Fu and LBP groups could arrest the AGS cell cycle in S phase (Figures 3(c) and 3(d)). Meanwhile, it was observed that although Nab-PTX + 5-Fu could stop the AGS cell cycle in the S phase, the proportion of the S phase increased compared with that of 5-Fu, and the G2/M phase decreased significantly compared with that of NAB-PTX (Figure 3(e)).

### 3.3. The Autophagy Signals Induced by Nab-PTX, 5-Fu, LBP, and Nab-PTX + 5-Fu Were Differentially Expressed

**Figure 4:**
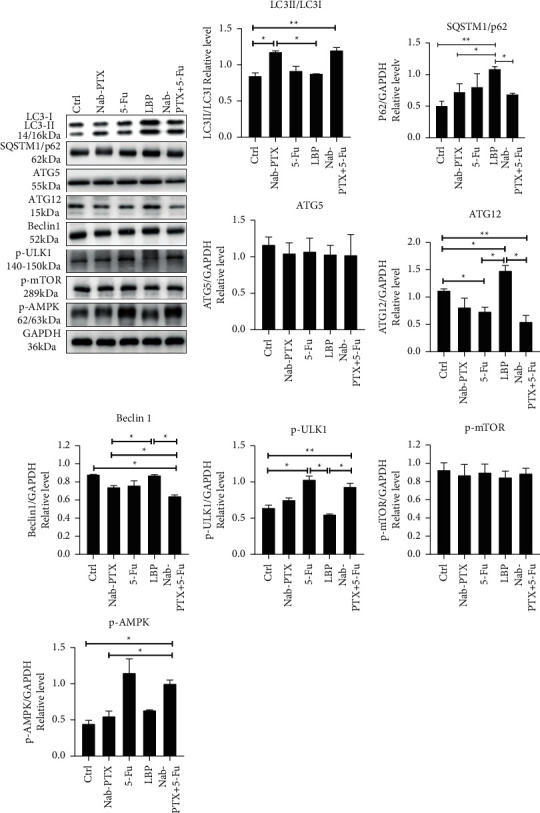
The effect of each group on autophagy of AGS cell line. Treatment of gastric cancer cells with Nab-PTX (0.2 *μ*mol/l), 5-Fu (2 *μ*mol/l), LBP (10 *μ*mol/l), and Nab-PTX + 5-Fu (0.2 *μ*mol/l + 2 *μ*mol/l) for 24 hours; after protein extraction, WB experiment was performed. WB results showed the protein expression of LC3II/LC3I, P62, Beclin-1, Atg12, Atg5, p-mULK1, and p-AMPK. All the above experiments were repeated at least three times independently. ^*∗*^*P* < 0.05, ^*∗∗*^*P* < 0.01.

To analyze the difference in autophagy signal expression when Nab-PTX, 5-Fu, LBP, and Nab-PTX + 5-Fu acted on AGS cells, we first detected the expression of LC3II/LC3I protein and SQSTM1/p62 by WB, and the results of Western blot autophagy signal protein showed (Figure 4): Nab-PTX and Nab-PTX + 5-Fu. The expression of LC3II/LC3I decreased, and SQSTM1/p62 increased in 5-Fu. The presentation of LC3II/LC3I in LBP decreased, but the absolute values of LC3II and LC3I increased, and the expression of SQSTM1/p62 increased significantly. Then, we detected the expression of Beclin-1 in each group. The presentation of Beclin-1 increased slightly in LBP but decreased in Nab-PTX, 5-Fu, and Nab-PTX + 5-Fu groups. At last, to clarify the changes in the autophagy signal pathway, we detected the expressions of ATG12, ATG5, p-ULK1, p-AMPK, and p-mTOR. The presentation of ATG12 in Nab-PTX, 5-Fu, and Nab-PTX + 5-Fu decreased, while that in LBP increased significantly. ATG5 and p-mTOR decreased slightly in each group. The expressions of p-AMPK and p-ULK1 in 5-Fu and Nab-PTX + 5-Fu increased significantly, while the terms of p-AMPK and p-ULK1 in Nab-PTX increased somewhat, while p-AMPK increased and p-ULK1 decreased significantly in LBP.

### 3.4. Nab-PTX, 5-Fu, LBP, and Nab-PTX + 5-Fu Induced Differential Expression of Apoptosis Signals

**Figure 5:**
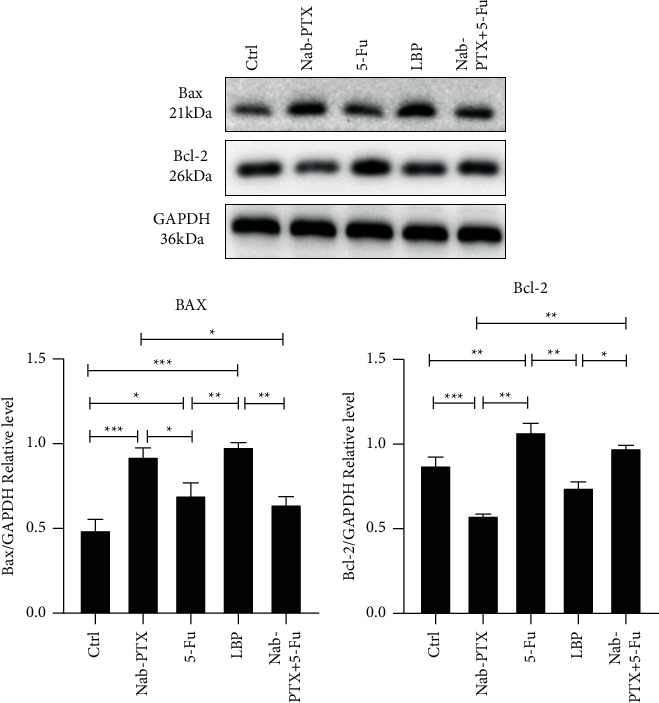
The effect of each group on apoptosis of AGS cell line. Treat gastric cancer cells with Nab-PTX (0.2 *μ*mol/l), 5-Fu (2 *μ*mol/l), LBP (10 *μ*mol/l), and Nab-PTX + 5-Fu (0.2 *μ*mol/l + 2 *μ*mol/l) for 24 hours. After extracting protein, the WB experiment was performed. WB results showed the protein expression of Bax and Bcl-2. All the above experiments were repeated at least three times independently. ^*∗*^*P* < 0.05, ^*∗∗*^*P* < 0.01, *P* < 0.001.

To analyze the differences in the expression of apoptosis signal proteins when Nab-PTX, 5-Fu, LBP, and Nab-PTX + 5-Fu act on AGS cells, the Western blot assay was used to detect the expression difference of Bax and Bcl-2. The results showed (Figure 5) that compared with the Ctrl group, Bax expression in each group was upregulated, and Bcl-2 in 5-Fu and Nab-PTX + 5-Fu.

## 4. Discussion

As we all know, Nab-PTX is a new solvent-free 130 nm nano-albumin-binding paclitaxel preparation developed by Celgene, an American biopharmaceutical company. The pharmacological characteristics of albumin paclitaxel are as follows: (1) albumin is reversibly transported through the body by binding to hydrophobic molecules and is quickly released on the cell surface. (2) The drug transmissibility is improved by passing components similar to plasma albumin into interstitial space through endothelial cells [19]. Nab-PTX has been widely used to treat breast cancer, non-small-cell lung cancer, and pancreatic cancer. The Japanese Guidelines for the Treatment of Gastric Cancer approved Nab-PTX to treat advanced gastric cancer in 2013. As a derivative of PTX, Nab-PTX has the same antitumor principle as PTX, which leads to G2/M phase stagnation by promoting tubulin polymerization and stabilizing microtubules, leading to abnormal mitosis of cancer cells and ultimately tumor cell death [20]. In this regard, the CCK8 cell proliferation test was conducted, and it was observed that Nab-PTX at a low dose could effectively inhibit the proliferation of AGS cell lines, and the inhibition rate increased with the increase of concentration and time. Additionally, flow cytometry was used to detect the cell cycle, and we also observed that NAB-PTX could effectively block the AGS cell cycle in the G2/M phase.

In our study, we found that 5-Fu and LBP could also inhibit the proliferation of AGS cell lines. In the cell cycle experiment, we found that both 5-Fu and LBP blocked the cell cycle in the S phase. However, the antitumor mechanisms of 5-Fu and LBP are not all the same. The tool of 5-Fu is to inhibit thymine nucleotide synthase and block the conversion of deoxy pyrimidine nucleotide to thymine nucleotide, thus interfering with DNA synthesis and playing an antitumor role [21, 22]. On the other hand, LBP produces alkylation, destroys DNA molecular structure, and makes cross-linking between bases, resulting in antitumor [23, 24]. Both 5-Fu and LBP ultimately interfere with DNA synthesis, thus arresting the cell cycle in the S phase. Interestingly, we found that Nab-PTX + 5-FU inhibited the AGS cell cycle in S and G2/M phases while effectively proliferating AGS cells and the proportion of the S phase was increased compared with 5-Fu. In contrast, the G2/M phase was significantly decreased compared with Nab-PTX. In the study of Passacantilli et al. [25], they found that Nab-PTX combined with 5-FU enhanced the accumulation of cells in the S phase, which was superior to the increase of cells accumulated in the S phase, leading to a sharp decrease of cells in G2 phase. It was speculated that Nab-PTX seemed to interfere with the early stage of the S phase. Further studies are needed to confirm this.

At present, the relationship between autophagy and cancer has always been a hot research topic, and there is no unified conclusion. Autophagy is now thought to play a protective role in cancer development, but autophagy plays the opposite protective role once cancer cells have formed. Autophagy also plays a double role in drug therapy of tumors [15, 26]. Therefore, the role of autophagy in the treatment of AGS cell lines by Nab-PTX, 5-Fu, LBP, and Nab-PTX + 5-Fu was further studied. We found that Nab-PTX + 5-Fu and Nab-PTX showed similarity in the downstream of autophagy: LC3II/LC3I expression increased and SQSTM1/P62 expression decreased, while Nab-PTX + 5-Fu expression was highly similar to 5-Fu expression in the upstream of autophagy. The expressions of p-AMPK and p-ULK1 were significantly increased, and the manifestation of Nab-PTX + 5-Fu and 5-Fu in the apoptosis signal was also very similar. The expression of Bcl-2 was significantly increased, while Bax was slightly increased. Studies have shown that excessive activation of autophagy can lead to type II programmed cell death, and excessive activation of autophagy leads to extreme degradation of mitochondria and other surviving molecules, ultimately leading to apoptosis [27, 28]. Combined with the apoptosis detection results of AGS cells treated by Nab-PTX, 5-FU, and Nab-PTX + 5-FU, namely, Nab-PTX + 5-Fu > Nab-PTX > 5-Fu, we speculated that NAB-PTX and NAB-PTX + 5-FU might promote the apoptosis of AGS cells through the activation of autophagy. The premise is that autophagy flows smoothly.

Beclin-1 is encoded by the BECN1 gene located on human chromosome 17Q21, and Beclin-1 plays a crucial role in regulating autophagic vesicle formation by activating PI3KC3 (also known as Vps34) and promoting the formation of PI3K complex (also known as Vps34 complex) [29]. Currently, it is recognized that BCL-2, an antiapoptotic protein of the Bcl family, is closely related to Beclin-1, which inhibits Beclin-1 activity through the BH3 domain of Beclin-1 [30, 31]. However, Beclin-1 is also regulated by a critical upstream target, ULK1, and AMPK seems to activate complexes involved in autophagy by phosphorylation of Beclin-1 [32]. Although 5-Fu and Nab-PTX + 5-Fu had similar expressions of p-AMPK, p-MTOR, and Bcl-2, the increase of Nab-PTX + 5-Fu was not as obvious as that of 5-Fu, which ultimately led to a significant decrease in Beclin-1 expression of Nab-PTX + 5-Fu. This also indicates that the regulation of autophagy is not a simple addition and subtraction relationship but a process of dynamic threshold change. It is worth mentioning that Beclin-1 often appears as a tumor suppressor gene in the process of tumor development. For example, BECN1 single allele deletion is commonly seen in breast cancer, ovarian cancer, and prostate cancer [33]. Beclin-1 expression in other tumors is lower than in normal tissues, but studies have shown that Beclin-1 is overexpressed in gastric cancer tissues. The high expression of Beclin-1 predicts a worse prognosis of gastric cancer [16, 33]. Thus, Nab-PTX + 5-Fu treatment of AGS cell lines resulted in a significant decrease in Beclin1, which seems to be a good sign in treating gastric cancer. In addition, further studies are needed to determine the cause for the difference in protein expression between Nab-PTX + 5-Fu, Nab-PTX, and 5-Fu in the upstream and downstream of autophagy, just like looking for a twisted valve between Nab-PTX autophagy and 5-Fu autophagy when the valve is twisted in a particular direction that results in the occurrence of Nab-PTX + 5-Fu autophagy.

Unlike the mechanism of action of Nab-PTX, 5-Fu, and Nab-PTX + 5-Fu, LBP seems to adopt another pathway. Our study found that the expression of LC3II/LC3I decreased in the LBP group, but the absolute value of LC3II and LC3I increased, and the expression of SQSTM1/p62 increased significantly. The presentation of ATG12 increased abnormally, suggesting that the block of autophagy flow might occur in the LBP group [34, 35]. A study on antitumor therapy for lung cancer [36] found that it induced intracellular ROS accumulation by blocking autophagy flux and enhanced the cytotoxicity of cisplatin and PTX to lung cancer cells. The possible mechanism is that hederagenin blocks the autophagy flux, induces the accumulation of mitochondrial ROS, and prevents the damaged mitochondria from being cleared through autophagy, thus enhancing the cytotoxicity of chemotherapy drugs. In addition, components of the molecular mechanism of autophagy also mediate unrelated functions of autophagy [37]. He Liu et al. [38] confirmed that ATG12 has an autophagy-independent role as an oncogene that promotes mitochondrial biogenesis. Due to ATG12 deficiency, mitochondrial biogenesis and cell bioenergetics are reduced, leading to tumor cell death. Swelling of cells and organelles and cytoplasmic bubbles were observed in the cells undergoing distension, which also proved that when ATG12 was deficient, energy deficiency caused damage to the cell ion pump. Coincidentally, Soren Mai et al. [39] found that overexpression of ATG12 leads to improvement of mitochondrial membrane potential, enhancement of ATP production, and antiapoptotic effect. Similar findings were also found in our experiment. LBP and NAB-PTX were identical in the expression of p-AMPK, p-MTOR, Atg5, Bax, Bcl-2, and other proteins. Still, the apoptosis experiment showed that the apoptosis rate of LBP was far lower than that of Nab-PTX, which may be related to the abnormal increase of ATG12 expression caused by LBP. It may also be related to autophagy flow blockage. Mitochondria may play an essential role in LBP-induced drug resistance of AGS cell lines. Notably, the expression level of Beclin-1 in the LBP group was similar to that in normal AGS cells without special treatment, suggesting that the high expression of Beclin-1 in gastric cancer cells does not seem to be beneficial for antitumor therapy.

In our study, differences in p-AMPK and p-ULK1 expressions were observed among the groups, and there was no significant difference in p-MTOR reaching the standard among the groups. Here, we focus on the significance of the differential expressions of p-AMPK and p-ULK1 in each group. First, let us take a look at the structure and function of AMPK: adenosine monophosphoric (AMP) activated protein kinase (AMPK)) is a heterotrimer *αβγ* complex that competitively binds AMP, ADP, and ATP to three sites in its *γ* subunit to sense the cellular energy state, of which AMP is the most significant [32, 40]. AMPK promotes autophagy not only directly through phosphorylation of ULK1 but also indirectly through the inactivation of mTORC1 [34]. In addition, AMPK is also a redox sensor and regulator. ROS-induced energy stress can strongly activate AMPK, independent of change in ADP, AMP, and ATP, and AMPK participation in ROS-triggered autophagy is considered critical to the survival of endothelial cells under stress conditions. AMPK also acts as a guardian of mitochondria [41].

Moreover, AMPK-induced autophagy is a crucial regulator of cell migration [42]. AMPK has been widely recognized as a promising pharmacological target, especially in treating diabetes, obesity, inflammation, and cancer [42, 43]. In short, AMPK's role in cells is complex. In our study, it was under such complex regulation that the autophagy downstream signals eventually changed significantly. Our experiment observed that LBP and NAB-PTX expressed similar amounts of p-AMPK and p-MTOR but significantly differed in the downstream effects on ULK1. Studies have shown that in addition to AMPK and mTOR regulation, ULK1 is also directly activated by TIP60 [44, 45]. More studies are needed to verify the role of this mechanism in LBP and gastric cancer cells.

Autophagy is involved in cancer growth, prevention, treatment, and drug resistance. At present, research on autophagy and immunotherapy is booming, and KRAS gene mutation leads to increased resistance to epidermal growth factor receptor- (EGFR-) targeted therapy [33]. Porrud et al. studied KRAS wild-type and mutant colorectal cancer cells (CRC) and cancer stem cells (CSCs), showing that 5-Fu can induce CRC apoptotic cell damage but upregulate CSCs autophagy, leading to drug resistance, but inhibition of this autophagy using 5-FU in combination with cetuximab, an anti-EGFR monoclonal antibody, increased the response of cancer stem cells to therapy [46]. As part of signaling pathways such as the RAS/MAPK pathway, BRAF is involved in cell differentiation, cell motility, and apoptosis [33]. In studies of melanoma, BRAF inhibitors can induce autophagy in different ways. BRAF combined with autophagy inhibitors may reverse resistance to BRAF inhibitors [47]. Furthermore, BRAF lncRNA may be another mechanism of tumor proliferation and tyrosine kinase inhibitor (TKI) escape in hepatocellular carcinoma (HCC) [48].

In conclusion, autophagy plays a complex role in treating gastric cancer. Nab-PTX, 5-Fu, LBP, and Nab-PTX + 5-Fu can inhibit cell proliferation, promote cell apoptosis to varying degrees, and induce differences in autophagy expression. The difference in autophagy of this antitumor drug may be related to the apoptosis induced by Nab-PTX. Nab-PTX induces more apoptosis than 5-Fu and LBP, which may be related to the increase in autophagy caused by Nab-PTX. Nab-PTX + 5-Fu induced more apoptosis than Nab-PTX and 5-Fu alone, which may be related to the rise and degree of autophagy induced by Nab-PTX + 5-Fu. In LBP, autophagy-mediated drug resistance may occur. It suggested that Nab-PTX has a better antitumor effect than 5-Fu and LBP in gastric cancer, and the combination of Nab-PTX + 5-Fu has more antitumor advantage. Based on the results of this experiment, for the treatment of gastric cancer, the combination drug still has a huge advantage over the single drug; although the role of autophagy in the antitumor effect of Nab-PTX, 5-Fu, and LBP is different, Nab-PTX is more effective than 5-Fu and LBP. It has antitumor advantages, suggesting that Nab-PTX is feasible for the treatment of gastric cancer and may also increase the efficacy and benefit of gastric cancer patients. At the same time, more studies are required to show the complex relationship between autophagy signal and apoptosis signal and autophagy signal and mitochondrial oxidative stress. In addition, our experiments are only carried out on AGS cells, leading to a certain deviation in our experimental conclusions in gastric cancer, so the findings of this experiment need to be verified on more cell lines.

## Data Availability

No data were used to support this study.

## Supplementary Materials

Supplementary Materials Here, we need to make the following additional remarks. (1) All the above WB results are true and reliable. In the WB Supplementary Materials provided, we attach the original film for explanation. (2) In the FACS experiment, we set the unstained/isotype control to ensure the authenticity of the experiment. Please refer to the FACS Supplementary Instructions for experimental group settings. (3) All data conform to the normal distribution (Gaussian) and allow parametric statistical testing; for this, we attach the original data of the experiments involved (SPSS analysis experiment raw data).
