# Supplementary material for: Albumin Paclitaxel Compared with 5-Penfluorouracil, Lobaplatin, and Albumin Paclitaxel Combined with 5-Penfluorouracil in the Treatment of Human Gastric Cancer Cell AGS Line Autophagy and Apoptosis
Source: Can J Gastroenterol Hepatol. 2022 Jun 10;2022:6015877. doi: 10.1155/2022/6015877 (PMC9205742; doi:10.1155/2022/6015877)
Supplement: Supplementary Materials — Here, we need to make the following additional remarks. (1) All the above WB results are true and reliable. In the WB Supplementary Materials provided, we attach the original film for explanation. (2) In the FACS experiment, we set the unstained/isotype control to ensure the authenticity of the experiment. Please refer to the FACS Supplementary Instructions for experimental group settings. (3) All data conform to the normal distribution (Gaussian) and allow parametric statistical testing; for this, we attach the original data of the experiments involved (SPSS analysis experiment raw data). [file 6015877.f1.zip › 6015877.f1/FACS Supplementary Instructions.docx]

| Number | Name | Fluorescent label  Annexin V | Nucleic acid dyes  PI |
| --- | --- | --- | --- |
| 1 | negative control | — | — |
| 2 | AGS cells(24h) | Annexin V | — |
| 3 | AGS cells(24h) | — | PI |
| 4 | Ctrl | Annexin V | PI |
| 5 | Nab-PTX | Annexin V | PI |
| 6 | 5-Fu | Annexin V | PI |
| 7 | LBP | Annexin V | PI |
| 8 | Nab-PTX+5-Fu | Annexin V | PI |

To ensure the accuracy of the experimental results, we employ unstained/isotype controls for FACS experiment, as the following table: All the above experiments were repeated three times using different batches of cells.
