# Supplementary material for: Albumin Paclitaxel Compared with 5-Penfluorouracil, Lobaplatin, and Albumin Paclitaxel Combined with 5-Penfluorouracil in the Treatment of Human Gastric Cancer Cell AGS Line Autophagy and Apoptosis
Source: Can J Gastroenterol Hepatol. 2022 Jun 10;2022:6015877. doi: 10.1155/2022/6015877 (PMC9205742; doi:10.1155/2022/6015877)
Supplement: Supplementary Materials — Here, we need to make the following additional remarks. (1) All the above WB results are true and reliable. In the WB Supplementary Materials provided, we attach the original film for explanation. (2) In the FACS experiment, we set the unstained/isotype control to ensure the authenticity of the experiment. Please refer to the FACS Supplementary Instructions for experimental group settings. (3) All data conform to the normal distribution (Gaussian) and allow parametric statistical testing; for this, we attach the original data of the experiments involved (SPSS analysis experiment raw data). [file 6015877.f1.zip › 6015877.f1/WB Supplementary Materials.pdf]

LC3II/LC3I

Maker | | | | | | Maker
15kDa | | | | | | 15kDa
10kDa | Ctrl | Nab-PTX | 5-Fu | LBP | Nab-PTX+5-Fu | 10kDa

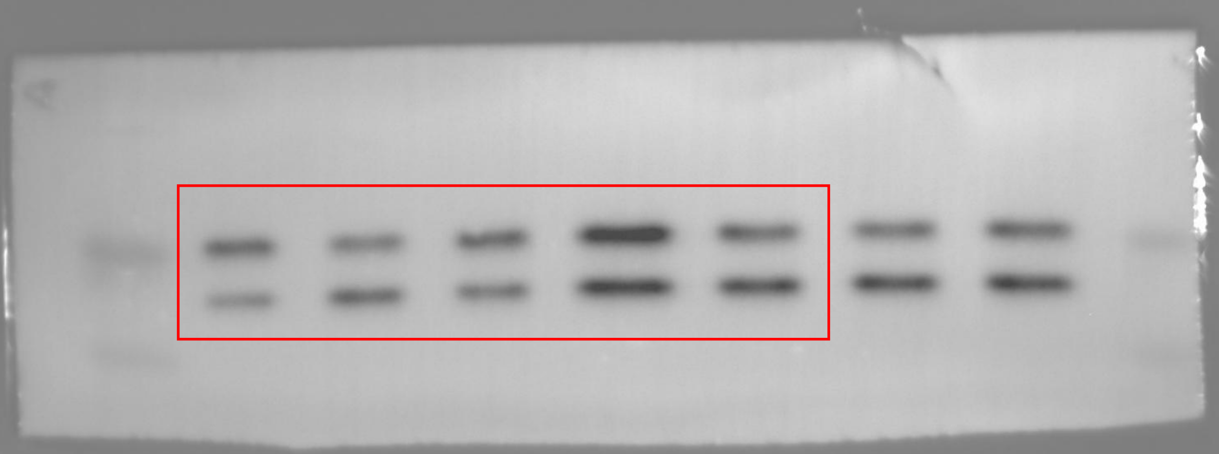

SQSTM1/p62

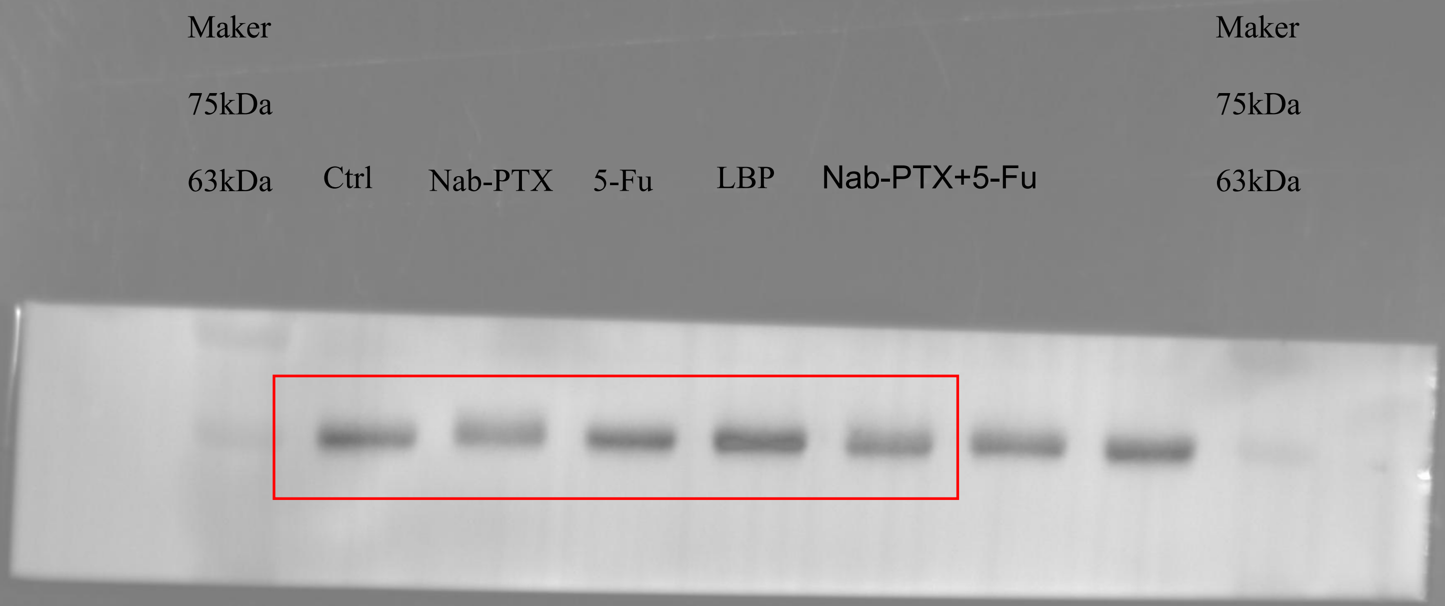
Maker
75kDa
63kDa
Ctrl
Nab-PTX
5-Fu
LBP
Nab-PTX+5-Fu
Maker
75kDa
63kDa

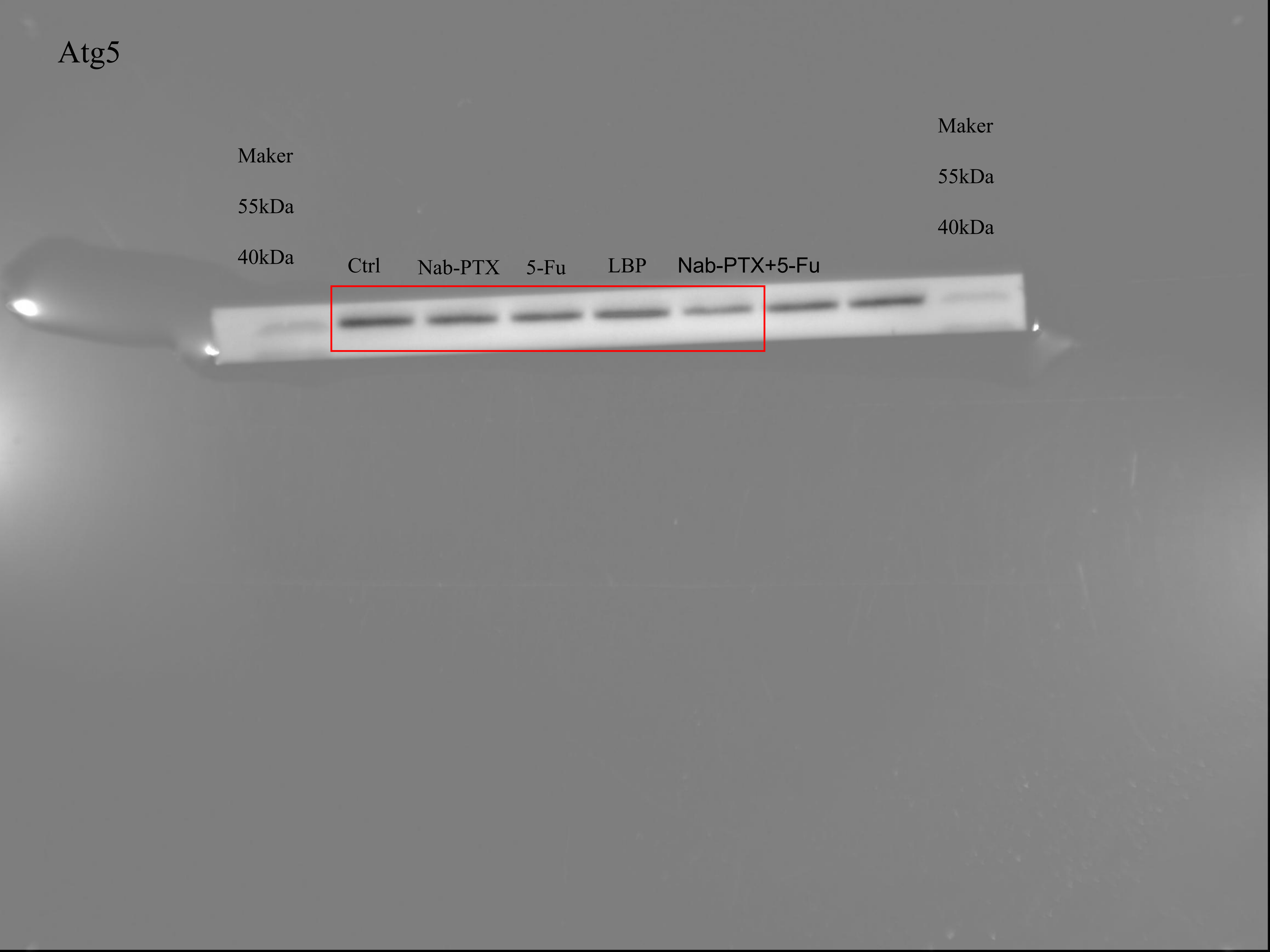
Atg5
Maker
55kDa
40kDa
Ctrl
Nab-PTX
5-Fu
LBP
Nab-PTX+5-Fu
Maker
55kDa
40kDa

Atg12

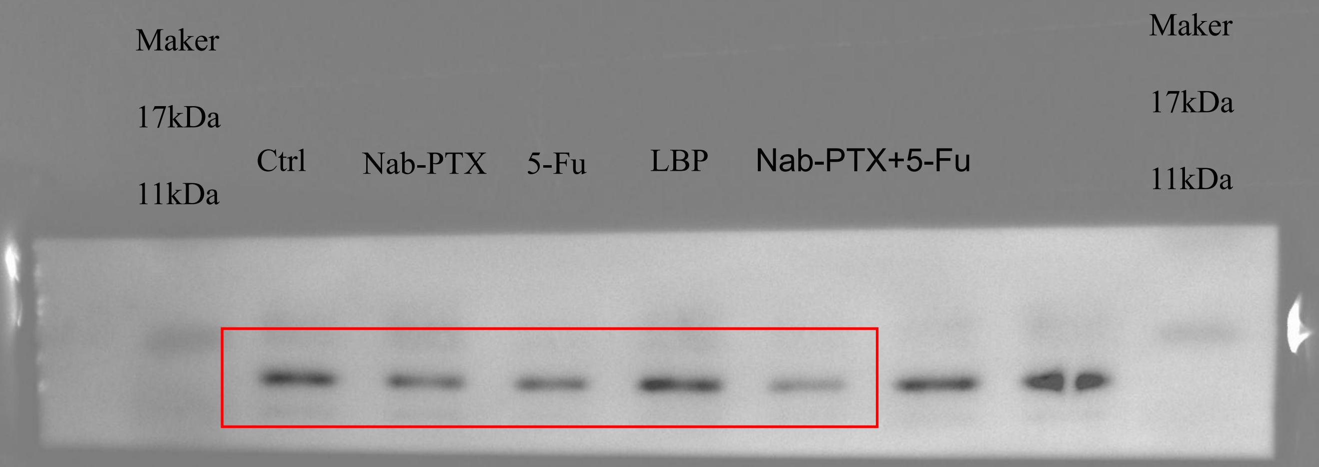

Maker
17kDa
11kDa
Ctrl
Nab-PTX
5-Fu
LBP
Nab-PTX+5-Fu
Maker
17kDa
11kDa

Beclin1

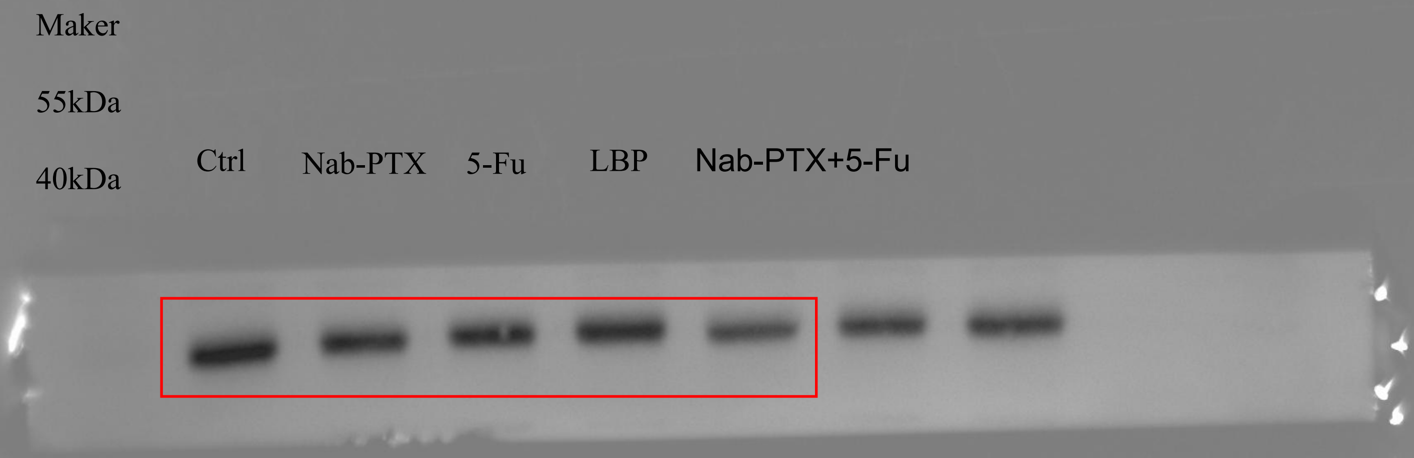

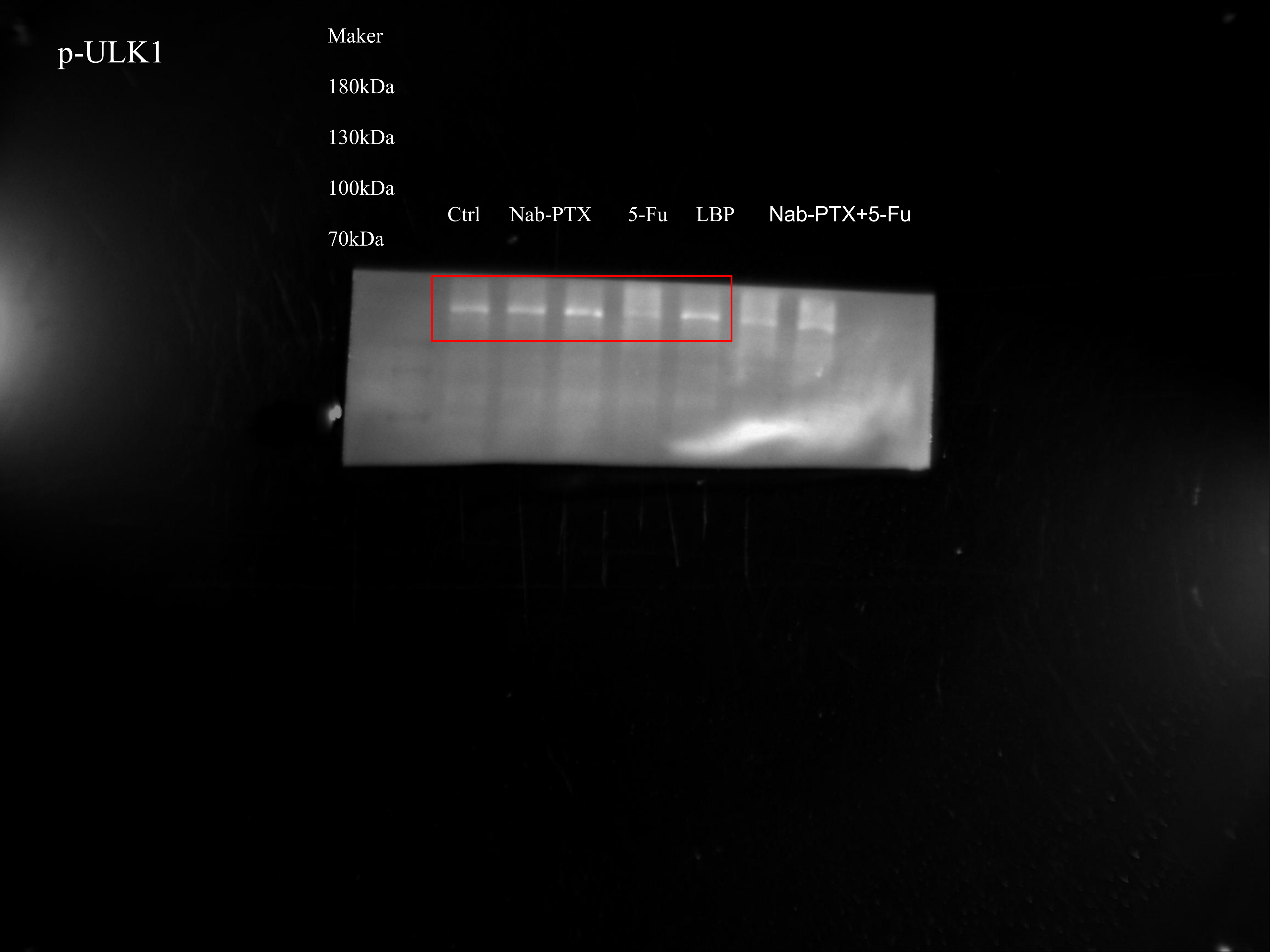
p-ULK1
Maker
180kDa
130kDa
100kDa
70kDa
Ctrl
Nab-PTX
5-Fu
LBP
Nab-PTX+5-Fu

p-mTOR

Ctrl Nab-PTX 5-Fu LBP Nab-PTX+5-Fu

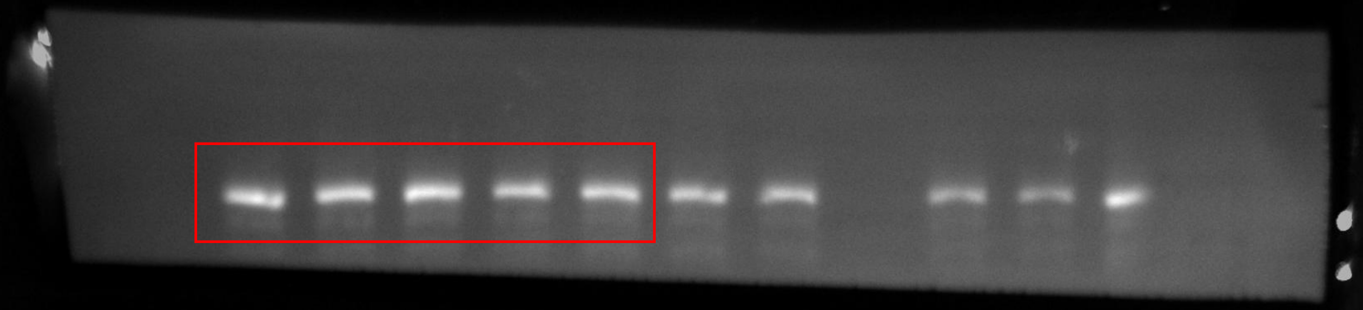

beyond the maker's
instructions

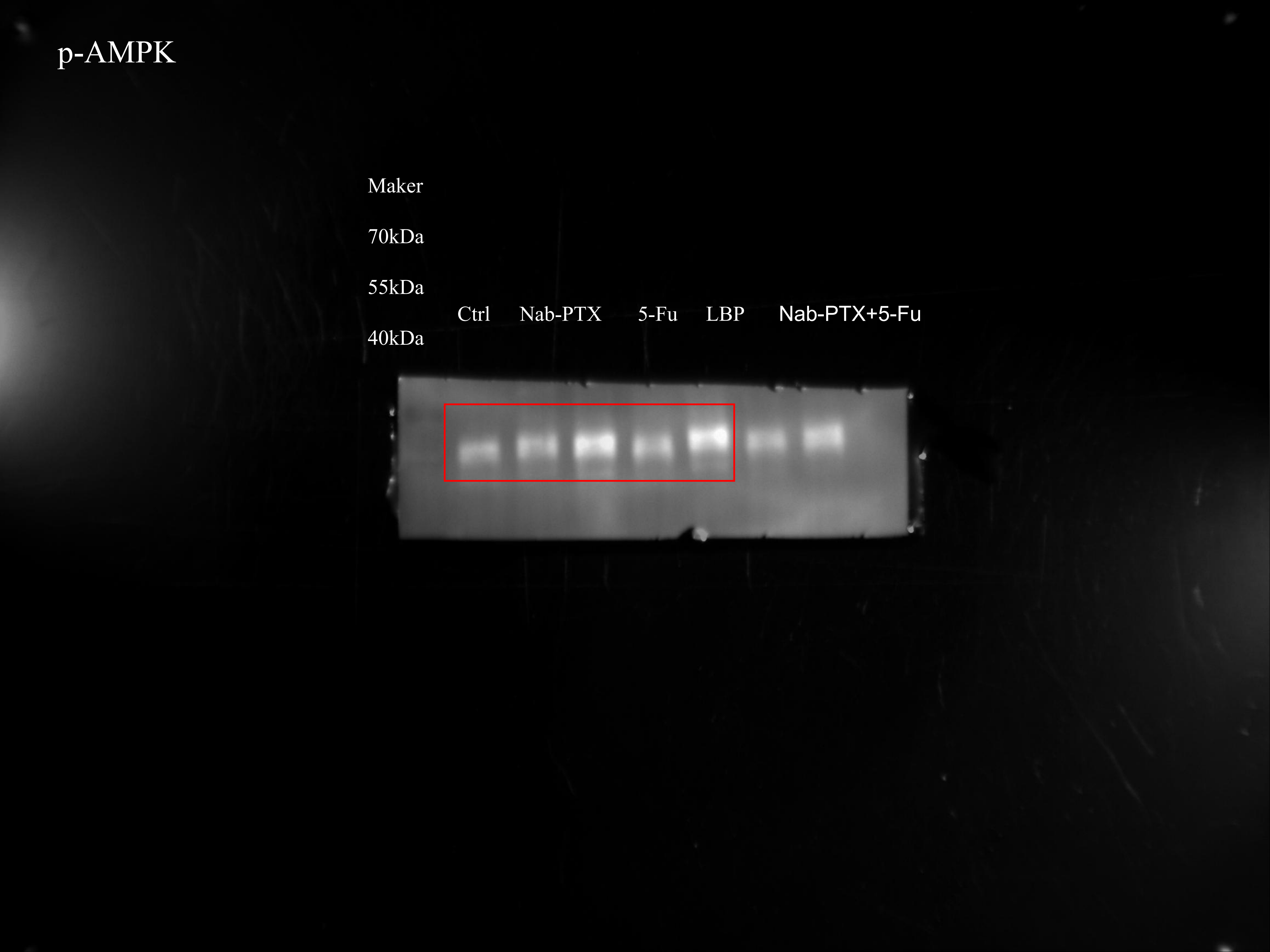
p-AMPK
Maker
70kDa
55kDa
40kDa
Ctrl
Nab-PTX
5-Fu
LBP
Nab-PTX+5-Fu

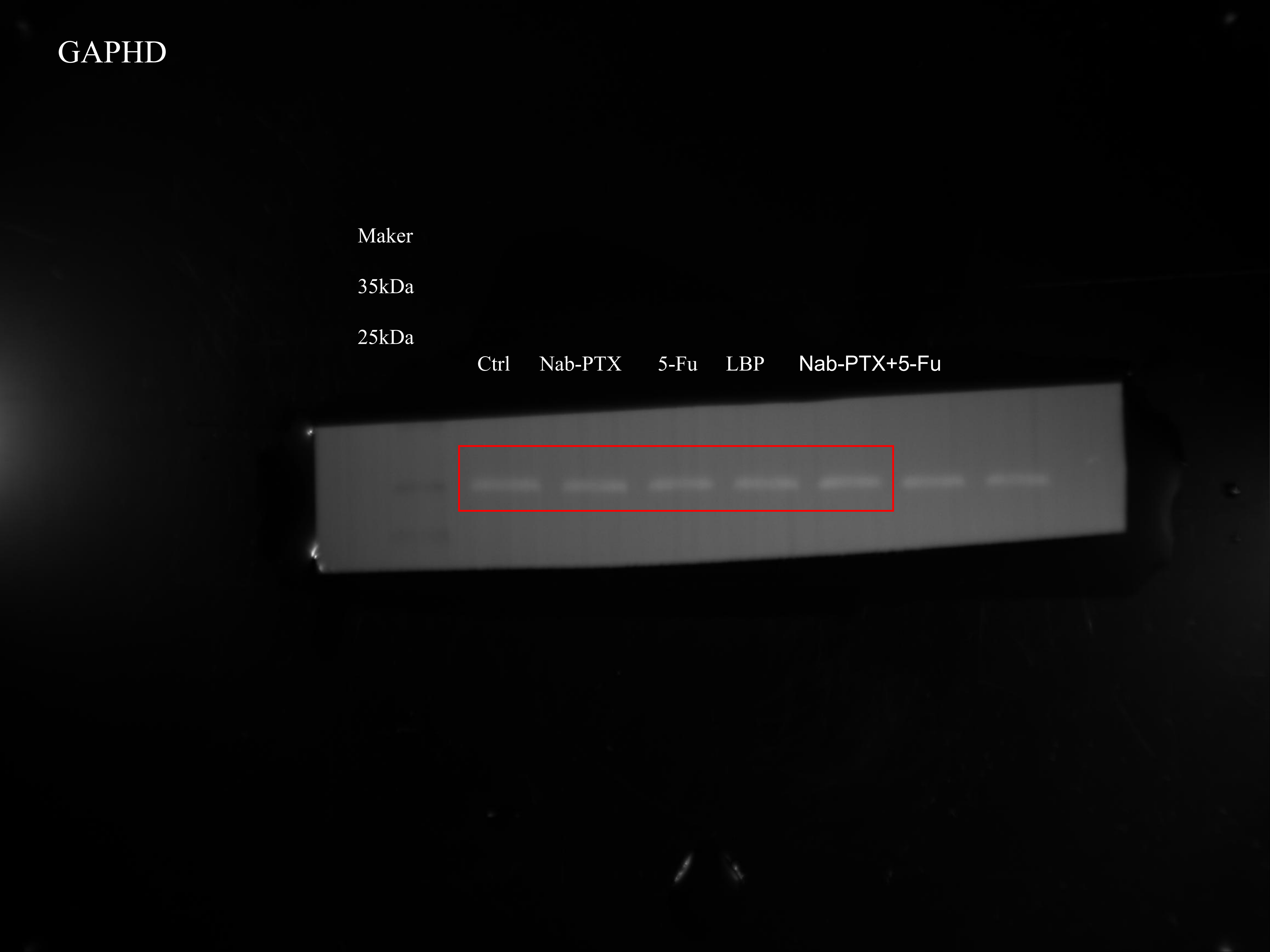
GAPHD
Maker
35kDa
25kDa
Ctrl
Nab-PTX
5-Fu
LBP
Nab-PTX+5-Fu

Bax

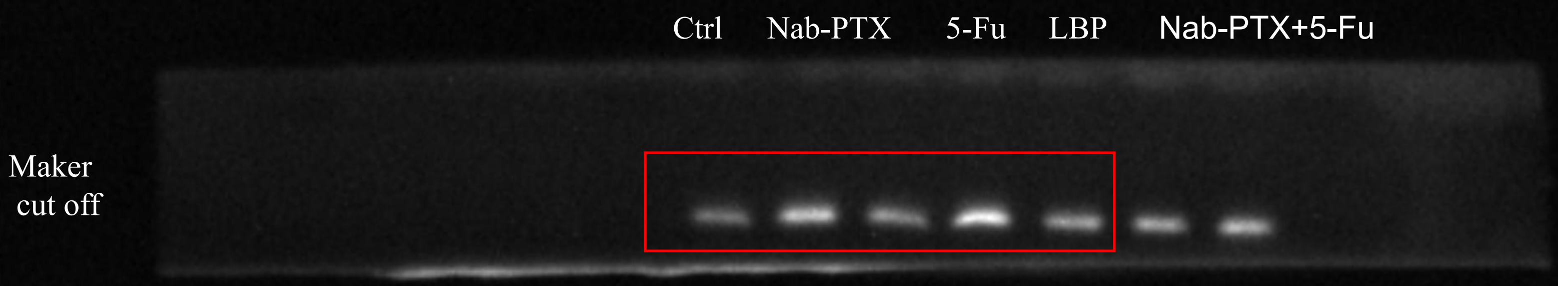

Bcl-2

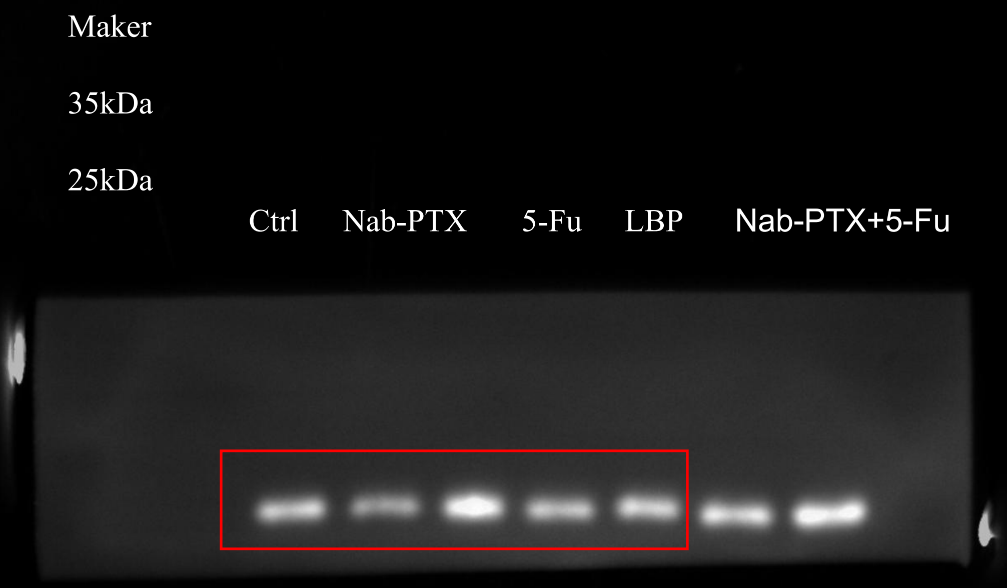

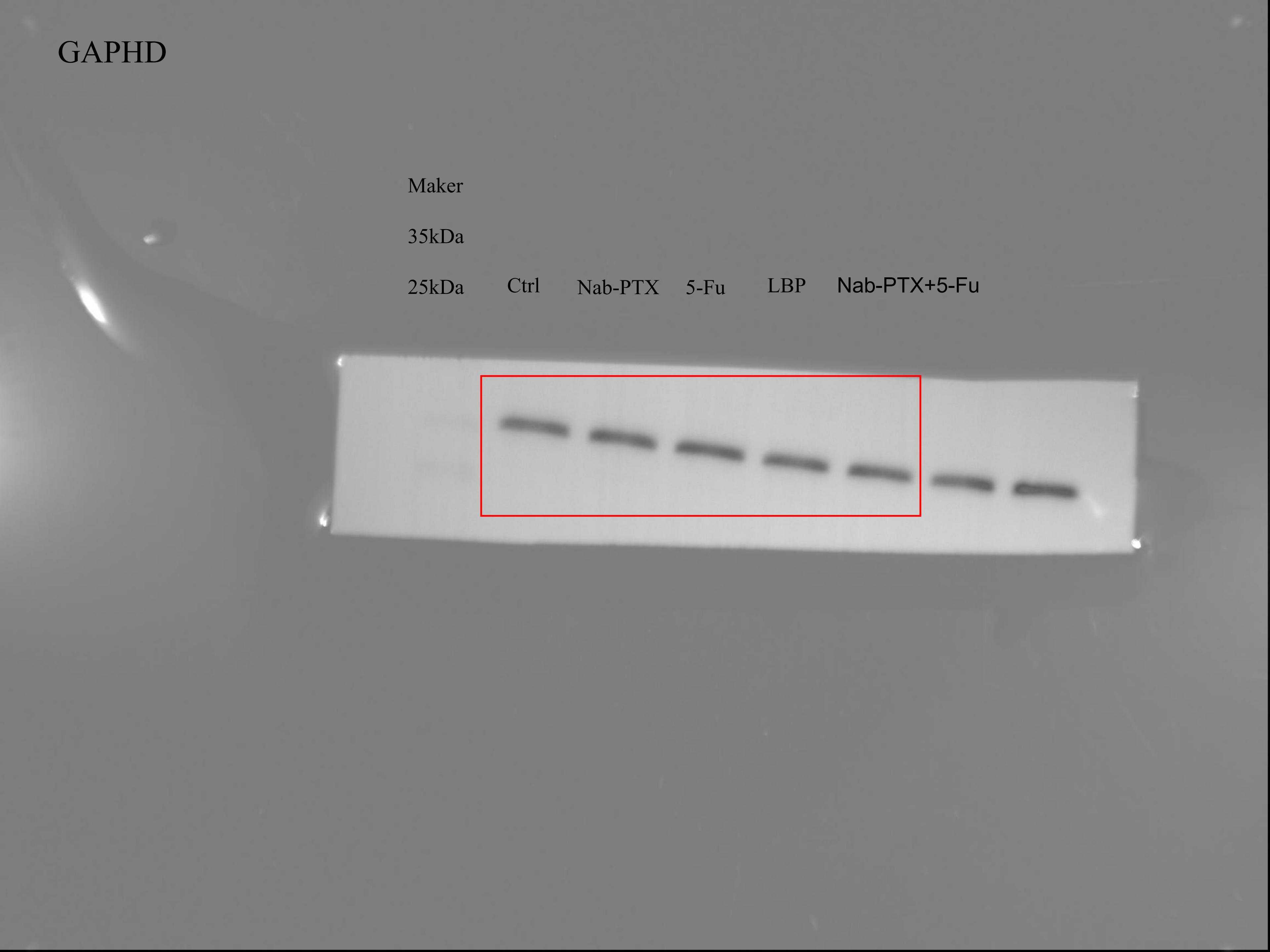
GAPHD
Maker
35kDa
25kDa
Ctrl
Nab-PTX
5-Fu
LBP
Nab-PTX+5-Fu
